# Structured evaluation of stress triggers in prehospital emergency medical care: An analysis by questionnaire regarding the professional groups

**DOI:** 10.1007/s00101-021-00968-x

**Published:** 2021-05-11

**Authors:** Hendrik Eismann, Lion Sieg, Thomas Palmaers, Vera Hagemann, Markus Flentje

**Affiliations:** 1 Department of Anaesthesiology and Intensive Care Medicine, Hannover Medical School, Carl-Neuberg-Straße 1, 30625 Hannover, Germany; 2 Faculty of Business Studies and Economics, University of Bremen, Enrique-Schmidt-Straße 1, 28359 Bremen, Germany

**Keywords:** Emergency ambulance systems, Performance improvement, Psychological conditions, Quality improvement, Stress management, Rettungsdienste, Leistungssteigerung, Psychische Rahmenbedingungen, Qualitätsverbesserung, Stressmanagement

## Abstract

**Background:**

Emergency medical services work in the environment of high responsibility teams and have to act under unpredictable working conditions. Stress occurs and has potential of negative effects on tasks, teamwork, prioritization processes and cognitive control. Stress is not exclusively dictated by the situation—the individuals rate the situation of having the necessary skills that a particular situation demands. There are different occupational groups in the emergency medical services in Germany. Training, tasks and legal framework of these groups vary.

**Objective:**

The aim of this study was to identify professional group-specific stressors for emergency medical services. These stress situations can be used to design skills building tools to enable individuals to cope with these stressors.

**Material and methods:**

The participants were invited to the study via posters and social media. An expert group (minimum 6 months of experience) developed a set of items via a two-step online Delphi survey. The experts were recruited from all professional groups represented in the German emergency medical service. We evaluated the resulting parameters for relevance and validity in a larger collective. Lastly, we identified stress factors that could be grouped in relevant scales. In total 1017 participants (paramedics, physicians) took part in the final validation survey.

**Results:**

After validation, we identified a catalogue of stressors with 7 scales and 25 items for EMT (Emergency Medical Technician) paramedics (KMO [Kayser-Meyer-Olkin criterion] 0.81), 6 scales and 24 items for advanced paramedics (KMO 0.82) and 6 scales and 24 items for EMS (Emergency Medical Service) physicians (KMO 0.82). For the professional group of EMT basic, the quality parameters did not allow further processing of the items.

Professional group-specific scales for EMT paramedics are “professional limitations”, “organizational framework”, “expectations” and “questions of meaning”. For advanced paramedics “appreciation”, “exceptional circumstances” and “legal certainty” were identified. The EMT physicians named “handling third parties”, “tolerance to ambiguity”, “task management” and “pressure to act”. A scale that is representative for all professional groups is “teamwork”. Organizational circumstances occur in all groups. The item “unnecessary missions” for EMT paramedics and “legal concerns with the application of methods” for advanced paramedics are examples.

**Discussion:**

Different stressors are relevant for the individual professional groups in the German emergency medical service. The developed catalogue can be used in the future to evaluate the subjective stress load of emergency service professionals. There are stressors that are inherent in the working environment (e.g. pressure to act) and others that can be improved through training (teamwork). We recommend training of general resistance as well as training of specific items (e.g., technical, nontechnical skills). All professionals mentioned items with respect to organizational factors. The responsible persons can identify potential for improvement based on the legal and organizational items. The EMT basic requires further subdivision according to task areas due to its variable applicability.

**Supplementary Information:**

The online version of this article (10.1007/s00101-021-00968-x) contains additional tables on stress factors of the surveyed groups. The article and supplementary material are available at www.springermedizin.de. Please enter the title of the article in the search field. The additional material can be found in the article under supplementary information.

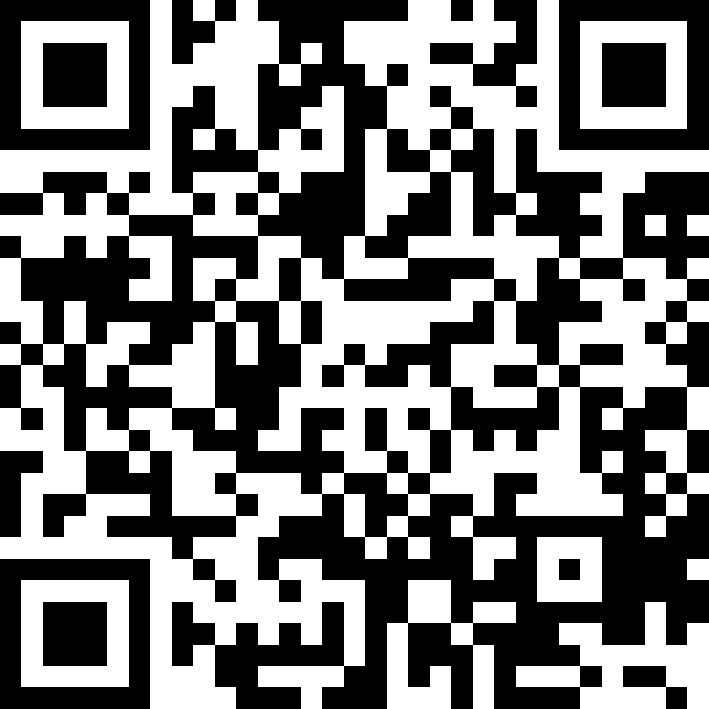

## Abstract (de)

**Hintergrund:**

Rettungsdienste arbeiten unter den Bedingungen von „High Responsibility Teams“ und müssen auch unter unvorhersehbaren Arbeitsbedingungen agieren. Stress tritt auf und hat potenziell negative Auswirkungen auf Aufgaben, Teamarbeit und Priorisierungsprozesse.

**Ziel der Arbeit:**

Das Ziel dieser Studie war es, berufsgruppenspezifische Stressoren für den Rettungsdienst zu identifizieren. Die identifizierten stressauslösenden Faktoren können genutzt werden, um Instrumente zum Aufbau von Fähigkeiten zu entwickeln, die es dem Einzelnen ermöglichen, mit diesen Stressoren umzugehen.

**Material und Methoden:**

Mittels einer zweistufigen Online-Delphi-Befragung, an der Teilnehmer aus allen im deutschen Rettungsdienst vertretenen Berufsgruppen teilnahmen, wurden Items entwickelt. Die daraus resultierenden Parameter wurden in einem größeren Kollektiv auf Relevanz und Validität überprüft. Schließlich wurden Belastungsfaktoren identifiziert, die in relevanten Skalen gruppiert werden konnten. Insgesamt nahmen 1017 Teilnehmer an der abschließenden Befragung teil.

**Ergebnisse:**

Nach der Validierung identifizierten wir einen Katalog von Stressoren mit 7 Skalen und 25 Items für Rettungsassistenten (KMO [Kayser-Meyer-Olkin Kriterium] 0,81), 6 Skalen und 24 Items für Notfallsanitäter (KMO 0,82) und 6 Skalen und 24 Items für Notärzte (KMO 0,82). Für die Berufsgruppe der Rettungssanitäter ließen die Gütekriterien keine weitere Bearbeitung der Items zu.

**Diskussion:**

Für die einzelnen Berufsgruppen im deutschen Rettungsdienst sind unterschiedliche Stressoren relevant. Der entwickelte Katalog kann in Zukunft zur Bewertung der subjektiven Stressbelastung von Rettungsdienstmitarbeitern eingesetzt werden. Der Rettungssanitäter bedarf aufgrund seiner unterschiedlichen Einsatzgebiete einer weiteren Untergliederung nach Aufgabenbereichen.

## Treten Sie in den Austausch

Diese Arbeit wurde für *Der Anaesthesist* in Englisch eingereicht und angenommen. Die deutsche Zusammenfassung wurde daher etwas ausführlicher gestaltet. Wenn Sie über diese Zusammenfassung hinaus Fragen haben und mehr wissen wollen, nehmen Sie, gern in Deutsch, über die Korrespondenzadresse am Ende des Beitrags Kontakt auf. Die Autor*innen freuen sich auf den Austausch mit Ihnen.

## Introduction and background

Emergency medical services (EMS) are typical representatives of so-called high responsibility teams (HRT). Their work environment is complex and demanding due to the dynamic and often unpredictable working conditions [11]. Under these conditions work-related stress and posttraumatic stress occur and the frequency of mishaps increases by a factor of 3 [4, 6]. Clinical performance and documentation appear to be vulnerable to the impact of acute stress [21]. Therefore, reducing stress in a healthcare team is a failure-avoidance strategy.

Chronic stress and critical incident stressors increase the risk of posttraumatic stress reactions and are associated with the development of cardiovascular disease and general morbidity [4, 22]. These are one of the main reasons of long-term absence [17]. Therefore, coping with stress also has an economic importance.

Stress is the state manifested by a specific syndrome which consists of all the nonspecifically induced changes within a biological system [28]. These reactions are not necessarily negative. The relationship between performance and stress has been known for a long time and is called the Yerkes-Dodson law [29]. Performance increases up to a point with incremental physiological or mental arousal. If the level of arousal continues to increase beyond this point, performance drops. Selye distinguished the two concepts “eustress” and “distress”. Eustress is described when the requirement is welcome. Distress is accompanied by negative consequences [27].

The present work deals with the identification of negative stressors (stress factors) depending on the professional group of EMS professionals. The perception and experience of stress is not wholly attributed to the situation the individual is in but also to the self-appraisal of having the necessary skills that the situation demands [20]. These proceed in three steps: firstly, (primary appraisal), the person judges the situation, secondly, the person decides if it is an impairment or a challenge (secondary appraisal). Subsequently, a re-evaluation is carried out and the results enter into a continuous learning process. Chronic stress occurs when a constant mismatch between personal skills and environmental needs occurs.

There are various occupational groups with different competences and responsibilities in the German emergency medical service (EMS) system. The task varies from an emergency medical technician (EMT basic, “Rettungssanitäter”) with a 3-month training (3 times 160 h training + a 40‑h examination week) to an EMS physician (“Notarzt”). In 2014 a professional profile advanced paramedic (“Notfallsanitäter”) with a 3-year training was established. The advanced paramedic replaces the EMT paramedic (“Rettungsassistent”) with a training of 2 years, who is no longer trained but such professionals are still working in the system. The advanced paramedic group has the qualification to administer medication and perform invasive procedures according to local protocols.

The aim of this study was to develop a catalogue of valid assessed stress-promoting situations regarding the professional groups in Germany. The catalogue will be usable to design skills building tools to enable individuals to cope with these stressors. Our hypotheses were: (a) relevant stress triggers in emergency service can be identified by a Delphi analysis and (b) the identified triggers are different depending on the profession.

## Study design and investigation methods

The ethics committee of the Hannover Medical School (no. 7256-2017) approved the study. All participants took part voluntarily without additional incentives. There is currently no established universal procedure for performing a Delphi analysis [13]. In comparison to other applications of the Delphi methodology in emergency medicine, our approach differed mainly with respect to cut-off limits and numbers of rounds [26]. The development of a suitable questionnaire to assess stress triggers in the EMS is divided into three parts.

### First part: questionnaire development

In the first step, we gathered information conducting an online-based Delphi analysis (eDelphi, electronic Delphi analysis) to identify relevant stress triggers.

For the Delphi analysis, a panel of EMS experts was constituted (15 experts per investigated professional group, 60 experts in total). The authors invited potential participants by means of posters and via social networks. At the time of the survey, all experts were actively working in EMS. We required at least 6 months of experience in the particular professional group (after training and certification) to also obtain stress triggers of young and mid-experienced EMS providers. All experts were asked the same eight initial questions (Table 1). The answers had to be given as a free text and they were unlimited. The online-based Delphi analysis and all further questioning were operated using the survey platform SurveyMonkey® (SurveyMonkey® Europe UC, Ireland). The initial questions of the Delphi analysis were developed by a local expert of emergency professionals and are shown in Table 1.

**Table 1:** Initial questions asked at the first round of the online-based Delphi analysis

	Original question text (German)	Translated question text (English)
Q1	Bitte nennen Sie uns Situationen aus ihrem Arbeitsalltag, die Sie als belastend empfinden	Please tell us about situations from your everyday work that you find stressful
Q2	Bitte nennen Sie uns Situationen aus Ihrem Arbeitsalltag, in denen Sie an Ihren erfahrenen Kollegen (>5 Jahre) eine erhöhte Belastung feststellen	Please state situations in your daily work routine in which you notice an increased burden on your experienced colleagues (>5 years)
Q3	Bitte nennen Sie uns Situationen aus Ihrem Arbeitsalltag, in denen Sie an Ihren unerfahrenen Kollegen (<5 Jahre) eine erhöhte Belastung feststellen	Please state situations in your daily work routine in which you notice an increased burden on your inexperienced colleagues (<5 years)
Q4	In welchen Situationen fühlen Sie sich in Ihrem Arbeitsalltag unwohl?	In which situations of your daily work routine do you feel uncomfortable?
Q5	Nennen Sie fünf Situationen, in denen Sie sich in Ihren Handlungen nicht sicher fühlen	Name five situations in which you do not feel secure in your actions
Q6	Gibt es Situationen in Ihrem Arbeitsalltag, bei denen Sie bei sich vermehrten Stress feststellen (erhöhte Schweißproduktion, Hitzewallungen, erhöhter Blutdruck, stockende oder stotternde Stimme, hastiges oder ungeduldiges Verhalten, innere Unruhe, konfliktreicher Umgang mit anderen Menschen)?	Are there situations in your everyday working life where you notice increased stress (increased sweat production, hot flushes, high blood pressure, faltering or stuttering voice, hasty or impatient behavior, inner restlessness, conflictual interaction with other people)?
Q7	Wenn Sie an den medizinischen Teil eines Einsatzes denken, was bereitet Ihnen dabei Stress?	When you think of the medical part of a mission, what causes you stress?
Q8	Wenn Sie an den medizinischen Teil eines Einsatzes denken, was denken Sie bereitet Ihren Kollegen dabei Stress?	When you think of the medical part of a mission, what do you think causes your colleagues stress?

Multiple answers from round 1 were summarized by the authors and a second survey was conducted subsequently [25]. The evaluation took place within the respective professional group. The participants received all answers tabulated during the first round of questions (the content from several of the answers was condensed by the authors prior to recirculation [25]) and were asked to select the most relevant items in two more rating rounds. The highest rated items were incorporated into the pre-test questionnaire. After receiving a broad consensus, we utilized the structured approach for a content analysis according to Mayring et al. [25]. The items were classified by the authors into self-defined categories, with a separate list for each professional group. The complication and the relevance of the items was reviewed in the next steps.

### Second part: pre-test questionnaire

The second step was to develop a pre-test questionnaire to identify reasonable items within the identified categories described above. The groups of experts were enlarged and the participants used a slider to rate the items on a scale from 0 (low) to 100 (very high) in the context of the personally felt stress. An example of the wording of the items is: “my perceived stress during confusing EMS missions is …”. We used this method to reach a higher validity and reliability of the test [5, 24]. The questionnaire contained a definition of stress and symptoms of stress. The participants were asked for their sex, age and the population of their main area of operation to differentiate between rural EMS and urban EMS. To recruit participants for this step, a link to the online questionnaire was send via email to the chief operating officers of EMS in Lower Saxony, Germany to distribute the links to all professionals. The survey was closed after 2 weeks.

The first phase of pre-test results analysis and item refinement were done by calculating the mean values and normal distributions of the items as well as checking their content validity. Questions with similar or shared content were then compared and from these similar items only those with a higher mean and normal distribution values were kept for further consideration. During the second phase, a reliability analysis was performed to check for internal consistency. From an economic perspective the final questionnaire should contain no more than 25 items per professional group. Consequently, a third phase of analysis was undertaken on the remaining items that rechecked the mean values, standard deviation and normal distribution, whilst ensuring that all response categories had been retained.

### Third part: development of the final questionnaire

In addition to the quality criteria of internal consistency, the final questionnaire was designed for a maximum completion time of 10 min. We analyzed all items with respect to content and discarded similar items or items with similar context in favor of items with better mean and better normal distribution (Table 2). Using the Kaiser-Guttmann criterion, the items were reclassified and named with categories. The analysis was carried out separately for each professional group. The final questionnaire resulted in 24 questions for EMT basic, 25 for EMT paramedics, 25 for advanced paramedics and in 23 for the prehospital emergency medicine physicians. To facilitate a valid analysis of the structure, we tested this result in a larger group of EMS professionals. A link to the survey was send via email, social networks (e.g. Facebook [Facebook Inc., Menlo Park, CA, USA] and Xing [New Work SE, Hamburg, Germany]), personal messaging services (e.g. iMessage [Apple Inc., Cupertino, CA, USA] and WhatsApp [Facebook Inc.; Menlo Park, CA, USA]) to all attainable EMS personnel throughout Germany. By performing an exploratory factor analysis, it was possible to deduce the presence of a few underlying latent variables from among the observations derived from the large number of items included in the questionnaire.

**Table 2:** Demographic data of the participant of the pre-test questionnaire

		*N*	%
Professional group	EMT basic	26	27.37
	EMT paramedic	18	18.95
	Advanced paramedic	24	25.26
	EMS physician	27	28.42
Sex	Female	20	21.05
	Male	75	78.95
Age (years)	Mean 35.37 (SD 10.03), (min. 20; max. 63)	95	–
Main area of operation	Rural EMS (population <50,000)	51	53.68
	Urban EMS (population >50,000)	44	46.32

*EMT* emergency medicine technician, *SD* standard deviation, *min* minimum, *max* maximum, *EMS* emergency medical service

## Statistics

All collected data were processed using SPSS 24 (IBM Corporation, Armonk, NY, USA). Data are presented as mean and standard deviation (normal distribution) or as median and interquartile range (non-normal distribution). A *p*-value <0.05 was considered statistically significant. The reliability of the scales was determined by Cronbach’s alpha. An exploratory factor analysis was performed. The condition for the feasibility of the factor analysis was verified by the Kaiser-Meyer-Olkin test (KMO). The Kaiser-Guttmann criterion was used to determine the number of factors, and an orthogonal varimax rotation was used to gain simplicity in factor interpretation during factor analysis [8].

## Results

### Delphi analysis

Initially, the emergency professionals mentioned for EMT basic 127, EMT paramedic 171, advanced paramedic 123 and physicians 139 items. After content summary and three rounds of the Delphi analysis, we found a variable number of relevant questions in the different professional groups (EMT paramedic 34 items, advanced paramedic 33 items and physicians 27 items). We identified the categories “organization”, “medicine”, “conflicts” and “communication” to cover all aspects of the consensus.

### Pre-test questionnaire

A total number of 95 EMS professionals completed the pre-test questionnaire (Table 3), 21.1% were female and the mean age was 35.4±10.0 years. The area of operation was rural EMS in 53.7% of the participants and in 46.3% urban environment, which was defined as a population greater than 50,000. This three-stage process of question refinement resulted in the elimination of 21 items in total (EMT paramedic 9 items, advanced paramedic 8 items, EMS physician 4 items) and the creation of a 23 item (EMS physician) and 25 item (EMT paramedic and advanced paramedic) final questionnaires (Table 2).

**Table 3:** Reliability of scales after completion of the pre-test questionnaire

Professional group	Scale	Pre-test items in total		Pre-test items after review with respect to content		Pre-test items after review with respect to reliability		Pre-test items after review with respect to SD and selectivity	
		*n*	Alpha	*n*	Alpha	*n*	Alpha	*n*	Alpha
EMT paramedic	Organization	11	0.715	10	0.651	7	0.757	7	0.757
	Medicine	8	0.795	9	0.819	8	0.840	8	0.840
	Conflicts	10	0.883	10	0.883	9	0.894	8	0.86
	Communication	5	0.108	4	0.454	2	0.811	2	0.811
Advanced paramedic	Organization	6	0.426	6	0.426	4	0.617	4	0.617
	Medicine	7	0.603	7	0.603	6	0.682	6	0.682
	Conflicts	15	0.792	15	0.792	13	0.717	11	0.824
	Communication	5	0.753	5	0.753	4	0.855	4	0.855
EMS physician	Organization	5	0.665	5	0.665	4	0.718	4	0.718
	Medicine	6	0.716	6	0.716	4	0.816	4	0.816
	Conflicts	11	0.833	11	0.833	10	0.855	10	0.855
	Communication	5	0.835	5	0.835	5	0.835	5	0.835

*n *number of questions, *alpha* Cronbach’s alpha, *SD* standard deviation, *EMT* emergency medicine technician, *EMS* emergency medical service

### Questionnaire validation

We received data of 206 EMT basic, 293 EMT paramedic, 292 advanced paramedic and of 226 EMS physician. Of the EMS professionals 19.3% were female, the mean age was 36.8 years (±11.1 years) and the mean experience in German EMS was 12.3 years (±9.5 years). The area of operation was defined by the number of inhabitants. From a rural EMS (population <50,000) we had 529 participants (46.2%), from urban EMS (population 50,000–150,000) 285 participants (24.9%) and from metropolitan EMS (population >150,000) 194 participants (17.0%). The breakdown by professional group is shown in Table 4.

**Table 4:** Demographic data of the participants of the final questionnaire

Professional group	*N*	%	Sex	*N*	%	Age (years)	Experience in EMS (years)	Main area of operation	*N*	%
EMT basic	206	18.0	Female	56	27.2	28.6 (±9.6)	5.2 (±6.6)	Rural EMS	108	52.4
	–	–	Male	149	72.3	–	–	Urban EMS	75	36.4
	–	–	–	–	–	–	–	Metropolitan EMS	21	10.2
EMT paramedic	293	25.6	Female	48	16.4	36.4 (±10.9)	12.2 (±8.9)	Rural EMS	169	57.7
	–	–	Male	243	82.9	–	–	Urban EMS	74	25.3
	–	–	–	–	–	–	–	Metropolitan EMS	47	16.0
Advanced paramedic	292	25.5	Female	43	14.7	37.2 (±8.9)	15.2 (±8.3)	Rural EMS	174	59.6
	–	–	Male	248	84.9	–	–	Urban EMS	69	23.6
	–	–	–	–	–	–	–	Metropolitan EMS	49	16.8
EMS physician	226	19.8	Female	74	32.7	44.4 (±9.7)	15.0 (±10.3)	Rural EMS	78	34.5
	–	–	Male	152	67.3	–	–	Urban EMS	67	29.6
	–	–	–	–	–	–	–	Metropolitan EMS	77	34.1

*EMT* emergency medicine technician, *EMS* emergency medical service

Rural EMS <50,000; urban EMS population 50,000–150,000; metropolitan EMS population >150,000

The exploratory factor analysis identified 6 factors and 25 items from the data, which accounted for 63.4% of the variance. Additionally, a factor loading of greater than 0.3 was calculated for the 25 items, which is considered relevant [8]. Sampling adequacy for the factor analysis was determined by use of the Kaiser-Meyer-Olkin (KMO) test, which returned a value of 0.927—KMO values greater than 0.8 are considered very good [8]. For the professional group of EMT basic, the necessary quality markers of validation phase were not sufficient, so we were not able to conduct a factor analysis (KMO 0.57). The factors, the resulting Cronbach’s alpha, the number of items per factor and the proportion of variance explained are shown in Table 5. The factors that did not meet the Kaiser-Guttman criterion were not included in the final analysis. Based on this criterion, a total of nine items were not included in the final list. Moreover, the original scales became redundant and required new categorization after factor analysis resulted in the creation of new item-groupings. The final lists of items of the individual professional group are shown in appendices 1–3.

**Table 5:** Exploratory factor analysis

	Factor number	Factor description	Cronbach’s alpha	Number of items	Explained variance (%)	KMO
EMT paramedic	1	Professional limitations	0.760	5	13.57	–
	2	Professional uncertainness	0.841	5	12.87	–
	3	Organizational framework	0.786	6	11.54	–
	4	Personal vulnerability	0.693	3	8.18	–
	5	Team performance	0.691	3	7.80	–
	6	Expectations	0.596	2	6.28	–
	7	Question of meaning	–	1	5.03	–
	–	–	–	–	65.30	0.81
Advanced paramedic	1	Professional uncertainness	0.858	7	15.14	–
	2	Team performance	0.803	6	14.01	–
	3	Appreciation	0.765	6	13.98	–
	4	Exceptional circumstances	0.468	2	8.06	–
	5	Legal certainty	0.832	2	7.89	–
	6	Personal vulnerability	–	1	6.27	–
	–	–	–	–	65.30	0.81
EMS physician	1	Pressure to act	0.862	7	17.28	–
	2	Handling third parties	0.794	5	11.36	–
	3	Team performance	0.820	3	11.31	–
	4	Organizational framework	0.697	5	8.74	–
	5	Tolerance to ambiguity	0.565	2	8.45	–
	6	Task management	0.766	2	7.89	–
	–	–	–	–	65.03	0.82

*KMO* Kaiser-Meyer-Olkin value, *EMT* emergency medicine technician, *EMS* emergency medical service

## Discussion

The aim of the study was to identify relevant stressors for the professional groups in the German EMS to have a basis for skill building tools to cope with the stressors. The development succeeded for the professional groups EMT paramedic (25 items), advanced paramedic (24 items) and EMS physician (24 items). The contents of scales and items vary according to the professional group, so this differential consideration is meaningful.

The work environment of emergency medical services supports the development of stress [7, 15]. Stressors resulting from action, roles and organizational structure are described [1]. We were able to develop detailed scales and items to describe the function-related stressors for EMT paramedic, advanced paramedic and EMS physician in Germany.

The developed factors show similarities to another developed questionnaire to the topic [7]. Not only specific medical challenges but also nontechnical items seem to be important. In contrast to a special keyword catalogue as developed for the EMS before, our analysis shows more general designations, e.g. “my perceived stress at rescue missions which needs complex tasks or planning, I sense …” [18]. When situations are specifically named, e.g., psychiatric and pediatric emergencies, they are in accordance with the literature [18].

The resulting first training goals for improvement with stress coping would be to improve the general handling of stress. Training of resilience could be superior to increasing the expertise in individual EMS scenarios. Resilience is a measure of the ability to cope with stress and thriving when faced with adversity [3]. Resilience is measurable and the connection to burnout was described for medical students [16]. There are various interventions to train resilience which are discussed in the literature [14]. Creating stable skills of resilience could be an option for coping strategies with unalterable stressors. Simple procedures, like having discussions with colleagues after the assignment, can reduce stress [2]. Implementation strategies should be pursued to increase the preforming of this debriefing.

For the professional group of EMT basic, the necessary quality markers of the validation phase were not sufficient, so we were not able to conduct an exploratory factor analysis (KMO 0.57). The EMT basic in Germany has a wide variety of application (disaster relief unit, transport of patients without vital threat, EMS). Each area of application has different working conditions. The EMT basic in a disaster relief unit often acts on a voluntary basis and is mainly pursuing another profession. Here the rarity of medical use in combination with the dimension of potential use (e.g. terror scenarios, huge accidents) can affect the stressors. The EMT basic in an ambulance has to monitor whether the condition of the patient is stable. The patient has been seen by a physician before. If the condition deteriorates the EMT basic has to organize advanced care. In the EMS the EMT basic has to assist at medical procedures and to drive the ambulance. They are supervised by paramedics at all times.

In order to answer hypothesis (b), the developed scales require qualitative consideration. All other three groups indicate team performance as a relevant scale for stressors. In addition to disagreements among colleagues, these stressors are the result of the situation in which rescue teams in Germany are working together as so-called ad hoc teams (teams with varying membership) in difficult situations. The physician and the team of the ambulance meet at the scene (rendezvous system) and may not know each other. For these ad hoc teams lack in cohesion and groupthink is described [23].

Our profession-specific catalogue enables the views of all participants to be evaluated with respect to the scale teamwork. As the EMT paramedic answer very general, to be stressed by disproportionate behavior of colleagues (e.g. inappropriate reaction), the advanced paramedic answer more specific in terms of communication and treatment planning (e.g. lack of team resource management).

The education of advanced paramedics includes patient treatment considering standard operation procedures (SOP) and team communication. The physicians have no specific training in this respect and are not bound to any SOP. The EMS physician feels pressure to act through the team (subverting of rescue team). These scales match the findings of a study which shows that conflicts between emergency physicians and advanced paramedics are more frequent than between other professional groups [9]. To improve these situations, training in communication and teamwork can be assumed to be more successful than training of medical content.

Other communication items (e.g. non-compliant physician in an emergency department) in respect of prehospital-hospital interfaces are also stressors in accordance with our previous work [9]. Here nontechnical skill training could be a success factor. As the group of people involved in prehospital patient care varies much (numerous hospitals and EMS with a great number of employees) it is a big challenge to solve.

The framework conditions of a medical emergency include stressors that cannot be changed. Hagemann et al. classified this working environment as high responsibility teams [12]. Among other specifics, work in the EMS is characterized by time pressure, taking responsibility for the lives of others, an irreversibility of measures taken and limited opportunities for rest of meal breaks. All three professional groups described the necessity of immediate medical treatment as a stressor.

The professional groups differ with respect to possibilities, application and implementation. The EMT paramedics mention missing treatment options (e.g. intravenous analgesia) and uncertainties of procedures applied. The scale professional limitations of the paramedic consists of seven items. In prior studies we found that advanced measures that have to be performed by paramedics, vary in training level and frequency of application [9, 10]. To reduce stress in this area training concepts should be integrated, which guarantee a high level of competence in all needed skills. The EMS physicians are responsible for the application of procedures in the complex work environment. The technical skills do not seem to be a relevant problem as the decision making in complex and dynamic situations is the main stressor.

German physicians are allowed to operate as EMS physicians after 2 years of clinical experience. Young physicians at that time are not used to make extensive decisions influencing the outcome of the critically ill patients (depending on the subspecialty). Simulation-based training might be an advantage for the physician and could reduce stress in real rescue missions.

All three professional groups mentioned items with respect to organizational factors. The EMT paramedics rate unnecessary missions as a factor. It is well known that EMS personnel want to apply what they have learned [19]. The advanced paramedics did not mention these circumstances. Since this profession was just created, it may be that the survey participants are not as long active in the job as other professional groups. This may interfere the preferences due to stress. The physicians often mainly work in a hospital environment so that this focus is not prioritized by them.

## Limitations

The general conditions of data protection forced us to make answering of the questionnaire not traceable. Theoretically multiple answers of the questionnaire by the same participant would be possible. Due to the high number of over 1000 participants, we tried to minimize this effect. The law for EMS in Germany is regulated at the level of the federal states. There may be regional peculiarities that are not reflected in the distribution of the results. The professional job profile of the advanced paramedic, which was introduced in 2014, is still novel and there are only brief experiences. Adjustments and changes in organizations in the next few years might change the conditions for stressors there.

## Conclusion

With our study, we were able to develop a professional-specific assessment catalogue for EMS professionals to assess stress factors. There are stressors that might be directly influenced by training, like technical skill and nontechnical skill training. There are also stressors that arise from the scope of EMS and cannot be changed. For this, the general resilience of the professionals would have to be strengthened. Our questionnaire is able to monitor the burden of stress on the professional groups and to evaluate training measures and interventions. It provides a basis for improving the working conditions for EMS professionals. Further research should accompany the implementation of the questionnaire. The job profile of the EMT basic requires intensive consideration to cater to this profession in term of stressors, too.

## Supplementary Information

ESM 1_Stress factors of the professional group EMT paramedic (“Rettungsassistent”)

ESM 2_ Stress factors of the professional group advanced paramedic (“Notfallsanitäter”)

ESM 3_Stress factors of the professional group EMS physician (“Notarzt”)

## Declarations

The study was approved by the ethics committee of the Hannover Medical School (no. 7256-2017). All participants agreed to participate in the study via the selection menu in the online questionnaire.
