# Supplementary material for: Structured evaluation of stress triggers in prehospital emergency medical care: An analysis by questionnaire regarding the professional groups
Source: Anaesthesist. 2021 May 11;71(4):291–8. doi: 10.1007/s00101-021-00968-x (PMC8986693; doi:10.1007/s00101-021-00968-x)
Supplement: Supplementary file 1 — ESM 1_Stress factors of the professional group EMT paramedic (“Rettungsassistent”) [file 101_2021_968_MOESM1_ESM.pdf]

Additional material to „Structured evaluation of stress trigger in prehospital medical care. An analysis by questionnaire regarding the professional groups". Eismann H., Sieg L., Palmaers T. et al. (2021) in *Der Anaesthesist*.
Article and supplementary material are available at www.springermedizin.de. Please enter the title of the article in the search field

---

Stress factors of the professional group EMT-paramedic ("Rettungsassistent")

Item number	Scale	Original item text (German)	Translated item text (English)
Q1	Professional limitations	„Meinen gefühlten Stress in Rettungsdiensteinsätzen bei Großschadenslagen (z.B. MANV) empfinde ich als…"	"My perceived stress at rescue missions at mass-casualty incidents, I sense..."
Q2		„Meinen gefühlten Stress in Rettungsdiensteinsätzen mit fehlender Möglichkeit der Analgesie durch Rettungsdienstpersonal empfinde ich als…"	"My perceived stress at rescue missions without the possibility of analgesia through EMS personnel, I sense..."
Q3		„Meinen gefühlten Stress in Rettungsdiensteinsätzen, bei denen man im Einsatz nicht mehr weiterweiß, empfinde ich als…"	" My perceived stress at rescue missions, where one feels helpless in the field, I sense..."
Q4		„Meinen gefühlten Stress in Rettungsdiensteinsätzen mit eigenständigem Medikamenteneinsatz empfinde ich als…"	"My perceived stress at rescue missions with self-contained use of medication, I sense..."
Q5		„Meinen gefühlten Stress in Rettungsdiensteinsätzen mit der Anlage eines intraossären Zugangs empfinde ich als…"	"My perceived stress at rescue missions with use of an intraosseous access, I sense..."
Q6	Professional uncertainness	„Meinen gefühlten Stress in Rettungsdiensteinsätzen bei der Durchführung von invasiven Maßnahmen bei nicht ganz klarer Indikation empfinde ich als…"	"My perceived stress at rescue missions with execution of invasive procedures with a vague indication, I sense..."
Q7		„Meinen gefühlten Stress bei der Diagnosefindung in Rettungsdiensteinsätzen bei unklaren Krankheitsbildern empfinde ich als…"	"My perceived stress at diagnosing vague clinical pictures during rescue missions, I sense..."
Q8		„Meinen gefühlten Stress in Rettungsdiensteinsätzen mit seltenen Notfallbildern empfinde ich als…"	"My perceived stress at rescue missions with rare emergency situations, I sense..."

Q9		„Meinen gefühlten Stress in Rettungsdiensteinsätzen bei Kindernotfällen empfinde ich als…“	"My perceived stress at rescue missions with pediatric emergencies, I sense..."
Q10		„Meinen gefühlten Stress beim Warten auf den Notarzt bei kritischen Patienten empfinde ich als…“	"My perceived stress at rescue missions while waiting for an emergency physician with a critical ill patient, I sense..."
Q11	Organisational framework	„Meinen gefühlten Stress bei vermehrter Dienstbelastung durch einen hohen Krankenstand empfinde ich als…“	"My perceived stress through the burden of an increased number of duties caused by a high status of employee´s illness, I sense..."
Q12		„Meinen gefühlten Stress im Rettungsdiensteinsatz im Schichtdienst empfinde ich als…“	"My perceived stress at rescue missions during shift work, I sense..."
Q13		„Meinen gefühlten Stress bei Alarmfahrten von Kollegen mit geringer Fahrpraxis empfinde ich als…“	"My perceived stress at emergency drives with colleagues having limited driving experience, I sense..."
Q14		„Meinen gefühlten Stress in Rettungsdiensteinsätzen bei fehlender Erholungszeit nach Nachtdiensten empfinde ich als…“	"My perceived stress at rescue missions and missing recovery time after night shift, I sense..."
Q15		„Meinen gefühlten Stress in Rettungsdiensteinsätzen kurz vor Dienstende empfinde ich als…“	"My perceived stress at rescue missions short before end of shift, I sense..."
Q16		„Meinen gefühlten Stress bei mehreren Nachtdiensten in Folge empfinde ich als…“	"My perceived stress at rescue missions at several night shifts in a row, I sense..."
Q17	Personal vulnerability	„Meinen gefühlten Stress im Rettungsdiensteinsatz beim Tod junger Patienten empfinde ich als…“	"My perceived stress at rescue missions with the death of young patients, I sense..."
Q18		„Meinen gefühlten Stress bei verbalen Angriffen auf das Rettungsdienstpersonal in Rettungsdiensteinsätzen empfinde ich als…“	"My perceived stress at verbal attacks against EMS personnel during rescue missions, I sense..."
Q19		„Meinen gefühlten Stress in Rettungsdiensteinsätzen mit aggressiven Patienten oder Angehörigen empfinde ich als…“	"My perceived stress at rescue missions with aggressive patients or family members, I sense..."
Q20	Team performance	„Meinen gefühlten Stress unangemessenen Reaktionen von Kollegen in Rettungsdiensteinsätzen empfinde ich als…“	"My perceived stress at inadequate reactions of colleagues during rescue missions, I sense..."
Q21		„Meinen gefühlten Stress in Rettungsdiensteinsätzen bei der Zusammenarbeit mit unmotivierten Kollegen empfinde ich als…“	"My perceived stress at rescue missions teamworking with unmotivated colleagues, I sense..."

Q22		„Meinen gefühlten Stress beim möglichen Vergessen eines wichtigen Versorgungsschrittes in Rettungsdiensteinsätzen empfinde ich als…“	"My perceived stress at potentially missing a critical step dealing with rescue missions, I sense..."
Q23	Expectations	„Meinen gefühlten Stress im Rettungsdiensteinsatz bei hoher Erwartungshaltung der Patienten bei Bagatellen empfinde ich als…“	"My perceived stress at rescue missions with high expectations of patients with bagatelles, I sense..."
Q24		„Meinen gefühlten Stress in Rettungsdiensteinsätzen bei der Diskussion mit Ärzten und Pflegepersonal in Krankenhäusern empfinde ich als…“	"My perceived stress at rescue missions with discussion with physicians and nurses in emergency departments, I sense..."
Q25	Question of meaning	„Meinen gefühlten Stress bei unnötigen Rettungsdiensteinsätzen empfinde ich als…“	"My perceived stress at unnecessary rescue missions, I sense..."
