# Supplementary material for: Structured evaluation of stress triggers in prehospital emergency medical care: An analysis by questionnaire regarding the professional groups
Source: Anaesthesist. 2021 May 11;71(4):291–8. doi: 10.1007/s00101-021-00968-x (PMC8986693; doi:10.1007/s00101-021-00968-x)
Supplement: Supplementary file 2 — ESM 2_ Stress factors of the professional group advanced paramedic (“Notfallsanitäter”) [file 101_2021_968_MOESM2_ESM.pdf]

Additional material to „Structured evaluation of stress trigger in prehospital medical care. An analysis by questionnaire regarding the professional groups". Eismann H., Sieg L., Palmaers T. et al. (2021) in *Der Anaesthesist*.

Article and supplementary material are available at www.springermedizin.de. Please enter the title of the article in the search field

---

Stress factors of the professional group Advanced paramedic ("Notfallsanitäter")

Item number	Scale	Original item text (German)	Translated item text (English)
Q1	Professional uncertainness	„Meinen gefühlten Stress im Rettungsdiensteinsatz bei fehlender Routine oder ungenügendem Training empfinde ich als…"	"My perceived stress at rescue missions with lack of routine or insufficient training, I sense..."
Q2		„Meinen gefühlten Stress im Rettungsdiensteinsatz bei individueller und nicht-leitliniengerechter Versorgung von Patienten empfinde ich als…"	"My perceived stress at rescue missions with non-guideline compliant care, I sense..."
Q3		„Meinen gefühlten Stress im Rettungsdiensteinsatz bei Unkenntnis bei der Bedienung medizinischer Geräte empfinde ich als…"	"My perceived stress at rescue missions and lack of knowledge of medical equipment, I sense..."
Q4		„Meinen gefühlten Stress im Rettungsdiensteinsatz bei der Durchführung von ungeübten Maßnahmen unter Zeitdruck empfinde ich als…"	"My perceived stress at rescue missions and application of untrained procedures under time pressure, I sense..."
Q5		„Meinen gefühlten Stress im Rettungsdiensteinsatz bei der Anwendung von seltenen/ neuen Maßnahmen empfinde ich als…"	"My perceived stress at rescue missions and the execution of rare or new procedures, I sense..."
Q6		„Meinen gefühlten Stress im Rettungsdiensteinsatz bei eigener Unsicherheit empfinde ich als…"	"My perceived stress at rescue missions and personal uncertainness, I sense..."
Q7		„Meinen gefühlten Stress im Rettungsdiensteinsatz beim Gefühl von Kontrollverlust im Einsatz empfinde ich als…"	"My perceived stress at rescue missions with a feeling of a loss of control, I sense..."
Q8	Team performance	„Meinen gefühlten Stress im Rettungsdiensteinsatz, wenn ältere Kollegen alles bagatellisieren, empfinde ich als…"	"My perceived stress at rescue missions when older colleagues trivialize everything, I sense..."
Q9		„Meinen gefühlten Stress bei der Zusammenarbeit mit schwierigen Teammitgliedern im Rettungsdiensteinsatz empfinde ich als…"	"My perceived stress while working together with difficult team members at rescue missions, I sense..."
Q10		„Meinen gefühlten Stress bei unstrukturierten Übergaben/	"My perceived stress while unstructured handover situation at hospitals, I sense..."

		Übernahmen in Kliniken empfinde ich als…“	
Q11		„Meinen gefühlten Stress im Rettungsdiensteinsatz bei fehlendem Team Resource Management empfinde ich als…“	"My perceived stress at rescue missions with missing team resource management, I sense..."
Q12		„Meinen gefühlten Stress im Rettungsdiensteinsatz bei schlechter Kommunikation im Team empfinde ich als…“	"My perceived stress at rescue missions with poor team communication, I sense..."
Q13		„Meinen gefühlten Stress im Rettungsdiensteinsatz bei fehlender Zusammenarbeit auf Augenhöhe empfinde ich als…“	"My perceived stress at rescue missions with missing team working at eye level, I sense..."
Q14	Appreciation	„Meinen gefühlten Stress bei fehlender Wertschätzung durch Vorgesetzte empfinde ich als…“	"My perceived stress at missing appreciation by superior, I sense..."
Q15		„Meinen gefühlten Stress im Rettungsdiensteinsatz mit Notärzten, die die Kompetenz des Rettungsdienstpersonals unterschätzen empfinde ich als…“	"My perceived stress at rescue missions with EMS-physicians that underestimate the competence of EMS personnel, I sense..."
Q16		„Meinen gefühlten Stress im Rettungsdiensteinsatz, wenn die Schnittstelle Krankenhaus auf einem schlechteren fachlichen Niveau arbeitet empfinde ich als…“	"My perceived stress at rescue missions with a hospital interface working on a poor professional level, I sense..."
Q17		„Meinen gefühlten Stress im Rettungsdiensteinsatz, wenn Notärzte vom Standard abweichen empfinde ich als…“	"My perceived stress at rescue missions with EMS-physicians deviate from standards, I sense..."
Q18		„Meinen gefühlten Stress im Rettungsdiensteinsatz bei ignorantem Auftreten anderer empfinde ich als: …“	"My perceived stress at rescue missions with ignorant behavior of others, I sense..."
Q19		„Meinen gefühlten Stress beim sich gegenüber Klinikpersonal rechtfertigen-müssen im Rettungsdiensteinsatz empfinde ich als…“	"My perceived stress at rescue missions while explaining myself as against the hospital personnel, I sense..."
Q20	Exceptional circumstances	„Meinen gefühlten Stress bei der Anwendung von chirurgischem Atemwegmanagement (Koniotomie) im Rettungsdiensteinsatz empfinde ich als…“	"My perceived stress at performing surgical airways (cricothyrotomy) during rescue missions, I sense..."
Q21		„Meinen gefühlten Stress im Rettungsdiensteinsatz bei komplexen Einsatzsituationen empfinde ich als…“	"My perceived stress at rescue missions with complex settings, I sense..."
Q22	Legal certainty	„Meinen gefühlten Stress im Rettungsdiensteinsatz bei komplexen Einsatzsituationen mit der Notwendigkeit einer sofortigen medikamentösen Intervention ohne ausreichende rechtliche Absicherung empfinde ich als…“	“My perceived stress in complex rescue mission situations with the need of urgent pharmacological treatment without sufficient legal protection, I sense…”
Q23		„Meinen gefühlten Stress im Rettungsdiensteinsatz bei rechtlichen	"My perceived stress at rescue missions with legal concerns during the care of patients, I sense..."

		Bedenken bei der Versorgung von Patienten empfinde ich als…“	
Q24	Personal vulnerability	„Meinen gefühlten Stress bei Angriffen auf das Team von Patienten oder Angehörigen im Rettungsdiensteinsatz empfinde ich als…“	"My perceived stress when patients or their relatives attack the team during rescue missions, I sense..."
