# Supplementary material for: Structured evaluation of stress triggers in prehospital emergency medical care: An analysis by questionnaire regarding the professional groups
Source: Anaesthesist. 2021 May 11;71(4):291–8. doi: 10.1007/s00101-021-00968-x (PMC8986693; doi:10.1007/s00101-021-00968-x)
Supplement: Supplementary file 3 — ESM 3_Stress factors of the professional group EMS physician (“Notarzt”) [file 101_2021_968_MOESM3_ESM.pdf]

Additional material to „Structured evaluation of stress trigger in prehospital medical care. An analysis by questionnaire regarding the professional groups". Eismann H., Sieg L., Palmaers T. et al. (2021) in *Der Anaesthesist*.

Article and supplementary material are available at www.springermedizin.de. Please enter the title of the article in the search field.

Stress factors of the professional group EMS-physician ("Notarzt") (EMS: emergency medical service)

Item number	Scale	Original item text (German)	Translated item text (English)
Q1	Pressure to act	„Meinen gefühlten Stress bei Rettungsdiensteinsätzen, die komplexe Handlungen oder Planungen notwendig machen empfinde ich als: …"	"My perceived stress at rescue missions which needs complex tasks or planning, I sense..."
Q2		„Meinen gefühlten Stress im Rettungsdiensteinsatz bei drohendem Verlust der Übersicht von komplexen Gesamtsituationen empfinde ich als: …"	"My perceived stress at rescue missions when faced with the threat of losing overview of complex situations, I sense..."
Q3		„Meinen gefühlten Stress bei unübersichtlichen Situationen im Rettungsdiensteinsatz empfinde ich als: …"	"My perceived stress at rescue missions with confusing situations, I sense..."
Q4		„Meinen gefühlten Stress im Rettungsdiensteinsatz bei der Entscheidung für oder gegen eine Intubation in Grenzfällen empfinde ich als: …"	"My perceived stress at rescue missions with borderline decisions for or against an intubation, I sense..."
Q5		„Meinen gefühlten Stress im Rettungsdiensteinsatz bei Patienten in akuter Lebensgefahr empfinde ich als: …"	"My perceived stress at rescue missions with patients in acute life-threatening situations, I sense..."
Q6		„Meinen gefühlten Stress im Rettungsdiensteinsatz bei Kindernotfällen oder kindlichen Traumata empfinde ich als: …"	"My perceived stress at rescue missions with pediatric emergencies or pediatric trauma, I sense..."
Q7		„Meinen gefühlten Stress im Rettungsdiensteinsatz bei Großschadenslagen empfinde ich als: …"	"My perceived stress at rescue missions in mass-casualty incidents, I sense..."
Q8	Handling third parties	„Meinen gefühlten Stress bei Übergabegesprächen an ältere oder "klugscheißende" Kollegen in Aufnahmeklinik im Rettungsdiensteinsatz empfinde ich als: …"	"My perceived stress during handover to older or "smart-ass" colleagues in the emergency department, I sense..."
Q9		„Meinen gefühlten Stress im Rettungsdiensteinsatz bei der Diskussion mit nicht-aufnahmewilligen Kliniken empfinde ich als: …"	"My perceived stress at rescue missions with discussions of hospitals not willing to admit, I sense..."

Q10		„Meinen gefühlten Stress im Rettungsdiensteinsatz bei der Unterwanderung der eigenen Entscheidungen durch Rettungsdienstpersonal empfinde ich als: …“	"My perceived stress at rescue missions with subverting my decisions by EMS personnel, I sense..."
Q11		„Meinen gefühlten Stress bei der Fremdscham bei inadäquat reagierendem Rettungsdienstpersonal im Rettungsdiensteinsatz empfinde ich als: …“	"My perceived stress at rescue missions with feeling embarrassed on behalf of inadequately acting EMS personnel, I sense..."
Q12		„Meinen gefühlten Stress im Rettungsdiensteinsatz bei unprofessionellem Ton des Notaufnahmepersonal empfinde ich als: …“	"My perceived stress at rescue missions with unprofessional behavior of emergency department personnel, I sense..."
Q13	Team performance	„Meinen gefühlten Stress im Rettungsdiensteinsatz mit jüngeren Kollegen auf einem schlechten Ausbildungsstand empfinde ich als: …“	"My perceived stress at rescue missions with young colleagues on a poor training level, I sense..."
Q14		„Meinen gefühlten Stress im Rettungsdiensteinsatz bei vermehrtem Arbeiten mit unerfahrenen Kollegen, die streng supervidiert werden müssen, empfinde ich als: …“	"My perceived stress at rescue missions with inexperienced colleagues who have to be strictly supervised, I sense..."
Q15		„Meinen gefühlten Stress im Rettungsdiensteinsatz mit schlecht ausgebildetem Rettungsdienstpersonal empfinde ich als: …“	"My perceived stress at rescue missions with poorly trained EMS personnel, I sense..."
Q16	Organisational framework	„Meinen gefühlten Stress im Rettungsdiensteinsatz bei Fremdbestimmung durch organisatorische Mängel empfinde ich als: …“	"My perceived stress at rescue missions with heteronomy due to organizational deficiencies, I sense..."
Q17		„Meinen gefühlten Stress bei der Zwangseinweisung nach PsychKG im Rettungsdiensteinsatz empfinde ich als: …“	"My perceived stress at rescue missions with the involuntary admission of psychiatric patients, I sense..."
Q18		„Meinen gefühlten Stress im Rettungsdiensteinsatz bei zunehmender Zahl von Einsätzen pro Schicht empfinde ich als: …“	"My perceived stress at rescue missions with increasing numbers of missions per shift, I sense..."
Q19		„Meinen gefühlten Stress im Rettungsdiensteinsatz bei unklaren Kompetenzen (z.B. Notaufnahmearzt will Verlegung in das Krankenhaus, als Notarzt ist man mit der Entscheidung aber nicht einverstanden) empfinde ich als: …“	"My perceived stress at rescue missions with unclear competences (e.g. physician of an emergency department wants a transfer to another hospital – EMS-physician does not agree with the decision), I sense..."
Q20		„Meinen gefühlten Stress im Rettungsdiensteinsatz bei der Diagnose einer Todesursache, wenn keine weiteren Informationen vorliegen empfinde ich als: …“	"My perceived stress at rescue missions in diagnosing a cause of death without further information, I sense..."
Q21	Tolerance to ambiguity	„Meinen gefühlten Stress im Rettungsdiensteinsatz bei korrektem Umgang mit gestresstem	"My perceived stress at rescue missions when dealing with stressed emergency department professionals,

		Notaufnahmepersonal ohne "selbst aus der Haut zu fahren" empfinde ich als: …“	without losing one´s patience, I sense..."
Q22		„Meinen gefühlten Stress im Rettungsdiensteinsatz bei aufgebrachten Angehörigen empfinde ich als: …“	"My perceived stress at rescue missions with furious relatives, I sense..."
Q23	Task management	„Meinen gefühlten Stress im Rettungsdiensteinsatz mit einem Teamleiter (z.B. Notarzt) ohne erkennbaren oder kommunizierten Plan empfinde ich als: …“	"My perceived stress at rescue missions with a team leader (e.g. emergency physician) without a recognizable or communicated plan, I sense..."
Q24		„Meinen gefühlten Stress im Rettungsdiensteinsatz mit unübersichtlicher Aufgabenverteilung empfinde ich als: …“	"My perceived stress at rescue missions with unclear task management, I sense..."
